# Should Heartbeats/Training Session Be Considered When Comparing the Cardiovascular Benefits of High-Intensity Interval Aerobic and Moderate-Intensity Continuous Training? A Critical Appraisal

**DOI:** 10.1186/s40798-020-00257-8

**Published:** 2020-07-15

**Authors:** Jhennyfer Aline Lima Rodrigues, Stella Vieira Philbois, Tábata de Paula Facioli, Ada Clarice Gastaldi, Hugo Celso Dutra de Souza

**Affiliations:** Department of Health Sciences, Exercise Physiology Laboratory, Ribeirão Preto Medical School, University of São Paulo, Av. Bandeirantes, 3900 - Vila Monte Alegre, Ribeirão Preto, SP 14049-900 Brazil

**Keywords:** Cardiovascular disease, Physical training, Heartbeat amplitude, Shear stress, Cardiorespiratory fitness

## Abstract

The prescription of physical training as a therapeutic measure in the treatment and control of chronic degenerative diseases, mainly cardiovascular disease and metabolic disease, is an increasingly used clinical approach, often preceding the pharmacological prescription. Despite the advances in exercise physiology and cardio functional performance in recent decades, the main challenge is to identify the most appropriate modality, intensity, and training volume for each pathophysiological situation. In this case, the superiority of high-intensity interval training (HIIT) over moderate-intensity continuous training (MICT) has been questioned, since many studies have shown similar results in the different physiological parameters evaluated, especially regarding cardiorespiratory fitness, cardiovascular autonomic control, and cardiac morpho functionality. The cause of conflicting results observed by different studies may be related to standardization, application, and comparison of the two protocols. HIIT would have a higher number of heartbeats compared to MICT, when maintaining high heart rate is disregarded. In this since, our hypothesis for the greatest gains in cardiorespiratory fitness and in the autonomic and cardiovascular adaptations promoted by HIIT is based on the higher volume of training performed as a function of the higher number of heartbeats per unit of time, since the intermittence was calculated based on a percentage of maximum heart rate or reserve heart rate. Nevertheless, the intermittency between the established heart rate percentages is not necessarily accompanied by the intermittent heart rate. Therefore, considering and matching the number of heartbeats performed per training session in both models seems to be a more appropriate way to compare the two training protocols.

## Key Points

1. Heartbeats/training session appears to be an important consideration when comparing the cardiovascular benefits of high-intensity interval training (HIIT) versus those of moderate-intensity continuous training (MICT).
2. The fact that HIIT training is associated with a higher number of heartbeats than MICT, when maintaining high heart rate is disregarded, might explain the superior cardiovascular benefits of HIIT.

## Introduction

The prescription of physical training as a therapeutic measure in the treatment and control of chronic degenerative diseases, mainly cardiovascular disease, is an increasingly used clinical approach, often preceding the pharmacological prescription. However, despite the advances in exercise physiology and cardio functional performance in recent decades, the main challenge is to identify the most appropriate modality, intensity, and training volume for each pathophysiological situation such as hypertension, diabetes, obesity, stroke, cancer, and others. The benefits of increased physical activity/exercise training, particularly as they lead to increased cardiorespiratory fitness, result in improved prognosis in a large spectrum of metabolic diseases and cardiovascular disease [1].

## High-Intensity Interval Training over Moderate-Intensity Continuous Training

In this sense, different options of physical training have been aimed to promote greater gains for the patient, mainly because supervised exercise is recommended as the first line of treatment for non-communicable chronic disease. This is the case with high-intensity interval training (HIIT). Therefore, knowing the cardiovascular responses and cardiovascular risk involved in performing this type of exercise is relevant for these patients [2]. HIIT consists of alternating periods of intensive aerobic exercise with periods of active or passive recovery [3–5]. Some authors suggest that the benefits of this training modality might be seen in a single session as well as in the long-term, outperforming gains compared to moderate-intensity continuous training (MICT) [6–8]. The main idea for the higher gains from HIIT pointed out by these authors is that during the high-intensity period, there is a high cardiac demand, close to maximum pumping capacity, and consequently, maximum oxygen uptake (VO2max) that may bring greater cardiovascular benefits when compared to MICT [5, 9, 10].

However, the superiority of HIIT over MICT has been questioned, since many studies have shown similar results in the different physiological parameters evaluated, especially regarding cardiorespiratory fitness (VO2peak and VO2max), cardiovascular autonomic control, and cardiac morpho functionality [11–14]. The cause of conflicting results observed by different studies may be related to standardization, application, and comparison of the two protocols. In this case, most studies compared the two modalities, HIIT and MICT, adjusting the training volume by time in minutes, since the daily session of HIIT was stipulated with a shorter time, aiming to equalize to the MICT energy expenditure, having as main reference the expected heart rate level for each intensity. However, our hypothesis for the greatest gains in cardiorespiratory fitness and in the autonomic and cardiovascular adaptations promoted by HIIT in some studies is based on the higher volume of training performed as a function of the higher number of heartbeats per unit of time, since the intermittence was calculated based on a percentage of maximum heart rate or reserve heart rate. Nevertheless, the intermittency between the established heart rate percentages is not necessarily accompanied by the intermittent heart rate. In this sense, although there is an intermittent speed and inclination in the treadmill as well as resistance in the cycle ergometer, the elevated heart rate corresponding to the period of greatest intensity remains almost the same during periods of low intensity. In summary, HIIT would have a higher number of heartbeats compared to MICT, when maintaining high heart rate is disregarded. This may explain the gains made by HIIT. Therefore, considering and matching the number of heartbeats performed per training session in both models seems to be a more appropriate way to compare the two training protocols.

On the other hand, there appears to be a superiority of HIIT with respect to vascular endothelium-dependent adaptations. In this case, HIIT would have a greater effect on blood pressure reduction, both after a single training session and over the long term [15–18]. This action would be due to the greater release of endothelium-derived relaxing factors, mainly nitric oxide, when compared to MICT. This vascular advantage is explained by the higher cardiac demand during the higher intermittent period, which leads to a higher elevation of systolic blood pressure, and consequently, increased shear stress in the vascular endothelium.

Additionally, it is noteworthy that HIIT has attracted new supporters and uses as a marketing strategy the shortened average time per session. Although some studies have reported its efficacy and safety [19–22], in some cases, HIIT protocols may induce cardiac risks, as their methodology employs alternating high-intensity heartbeat interspersed with “passive” recovery periods. However, during this passive phase, there is only a slight reduction in heart rate, especially in those individuals with low cardiorespiratory fitness. This procedure might induce risks since venous return is impaired by interruption of muscle pump stimulation (gastrocnemius and soleus). This phase may predispose to major cardiovascular complications resulting from the abrupt drop in cardiomyocyte oxygenation due to the reduction in cardiac index.

## Conclusion

In summary, our comments emphasize that considering and matching the number of heartbeats performed per training session in both models seems to be a more appropriate way to compare the two training protocols HIIT and MICT. Furthermore, we highlighted the procedures and care needed to prescribe HIIT in order to obtain greater gains and safety for the general population. Finally, all patients should also be examined by a cardiologist, who can assess the clinical condition, medication use, and the results of cardiological assessments performed prior to the start of any physical exercise protocol.

## Data Availability

Not applicable.

## Abbreviations

HIIT: High-intensity interval training
MICT: Moderate-intensity continuous training
